# Approach control. Stereoelectronic origin of geometric constraints on N-to-S and N-to-O acyl shifts in peptides†
†Electronic supplementary information (ESI) available: B3LYP/6-311++G(d,p) energies and Cartesian coordinates of all species; M06-2X/6-311++G(d,p) energies and B3LYP/6-311++G(d,p) free energies of **1**, **2**, and **3**, X = O; calculations of modified Bürgi–Dunitz angles *φ*′_BD_; ball-and-stick models of calculated transition states for N-to-X acyl transfer, MOs (HOMO-1) for **2**^‡^. See DOI: 10.1039/c7sc04046f


**DOI:** 10.1039/c7sc04046f

**Published:** 2018-01-08

**Authors:** Neal K. Devaraj, Charles L. Perrin

**Affiliations:** a Dept. of Chemistry & Biochemistry , Univ. Calif. San Diego , La Jolla , CA 92093-0358 , USA . Email: cperrin@ucsd.edu

## Abstract

Intramolecular N-to-S or N-to-O acyl shifts in peptides are of fundamental and practical importance, as they constitute the first step in protein splicing and can be used for the synthesis of thioester-modified peptides required for native chemical ligation. It has been stated that the nucleophile must be positioned *anti* to the carbonyl oxygen, as in a *cis* amide. Despite the importance of such reactions, an understanding of this geometric restriction remains obscure. Here we argue that the empirical requirement for positioning the nucleophile is a stereoelectronic effect arising from the ease of approach of the nucleophile to a carbonyl group, not ground-state destabilization. DFT calculations on model amides support our explanation and indicate a significant decrease in both the transition-state energy and the activation energy for a *cis* amide. However, the approach of the nucleophile must be *anti* not only to the carbonyl oxygen but also to the nitrogen. The direction of approach is expressed by a new, modified Bürgi–Dunitz angle. Our data shed light on the mechanisms of acyl shifts in peptides, and they explain why a *cis* peptide might be required for protein splicing. The further implications for acyl shits in homoserine and homocysteine peptides and for aldol condensations are also considered.

## Abstract 2

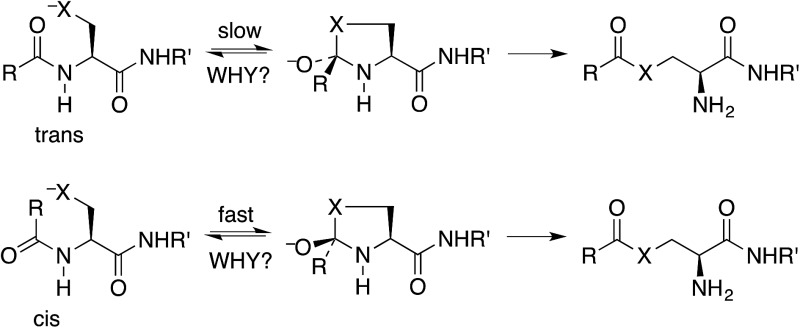
Intramolecular N-to-S or N-to-O acyl shifts in peptides are of fundamental and practical importance, as they constitute the first step in protein splicing and can be used for the synthesis of thioester-modified peptides required for native chemical ligation.

## Introduction

Protein splicing is the posttranslational excision of an internal polypeptide sequence, the intein, followed by ligation of the C-terminal and N-terminal segments, thereby generating the spliced extein.^1^ For standard class I inteins (which have a nucleophilic amino acid as the *N*-terminal residue),^2^ the initial step in this process utilizes an N-to-S or N-to-O acyl shift in a cysteine or serine residue, to produce a thioester or ester that is more reactive than the original amide toward nucleophilic attack.^3^ This process has been exploited for several important protein-engineering applications, such as expressed-protein ligation and recombinant-protein purification.^4^ The intramolecular N-to-S acyl shift reaction is also valuable for synthesizing peptide thioesters for native chemical ligation.^5^ This technique has become increasingly popular for such tasks as coupling to an alanyl or serinyl peptide by selective deselenization,^6^ synthesizing phospholipids,^7^ and generating a mixture of peptides in a dynamic exchange equilibrium.^8^

**Scheme 1:**
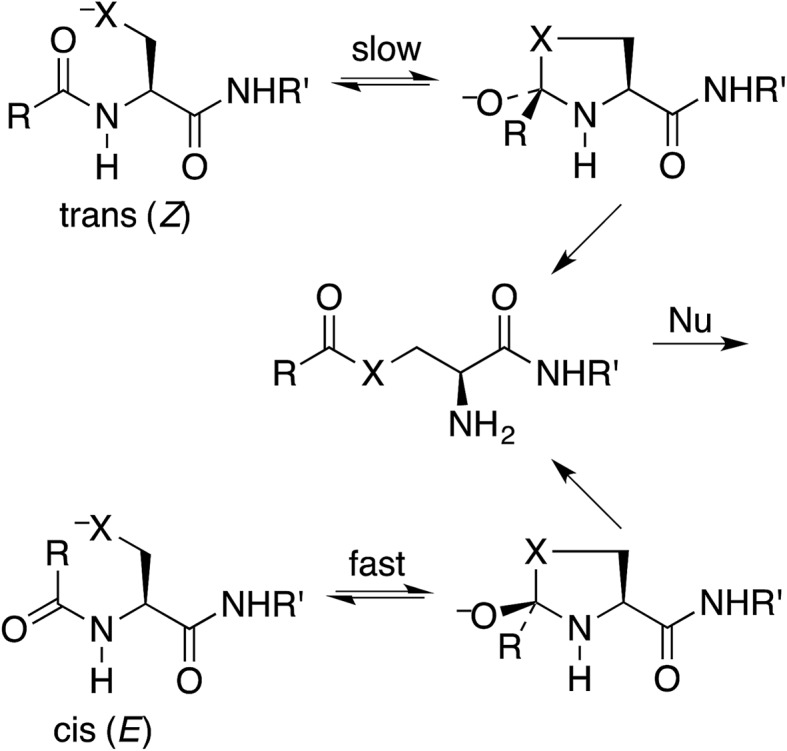
Activation of an acylcysteine or acylserine residue of a peptide or protein by N-to-S (X = S) or N-to-O (X = O) acyl shift, which is faster for a *cis* peptide.

It has been stated that the nucleophilic S or O must be positioned *anti* to the carbonyl oxygen for the N-to-S or N-to-O acyl shift to take place.^9^ Thus the shift is faster for a *cis* (*E*) amide, even though the product ester is the same from either (Scheme 1). Structural studies on class I inteins have illuminated the details of the initial acyl shift in proteins.^10^ In many cases the scissile peptide bond is found to be distorted,^11^ or in a *cis* conformation.^12^ In synthetic peptides a nucleophile *anti* to the carbonyl oxygen can be achieved with the *cis* stereoisomer of a secondary amide, such as an *N*-alkyl cysteine or serine,^13^ or else a bis(mercaptoethyl)amide.^14^ It should be noted that the reverse reaction, an S-to-N acyl shift, is key to the synthesis of proteins by native chemical ligation.^15^ In this case there is no stereochemical constraint imposed by the thioester.

The issue we address is the requirement that the nucleophilic S or O must be *anti* to the carbonyl oxygen. Among the suggestions that we reject are the steric effect that destabilizes the *cis* amide and the more similar interconversion rate between *cis* and *trans* in an *N*-alkyl amide.^16^ These explanations violate the Curtin–Hammett principle,^17^ which states that the relative rates and the product distribution depend only on the relative energies of the two transition states and not on the equilibrium between the reactants. Also, although ground-state destabilization is well established for some enzyme catalysis,^18^ this cannot explain the greater reactivity of *cis* amides. The fallacy is the assumption that the transition state is the same for *cis* and *trans* amides, made implicitly,^19^ whereas the steric repulsion that destabilizes a *cis* amide is still present in its transition state for cyclization, the key first step in the acyl shift. Therefore the destabilization of a *cis* amide is irrelevant. Nor does invoking the power of the enzyme to twist the amide bond^20^ or to *N*-protonate a twisted amide^21^ explain why a *cis* amide is more reactive.^22^

These acyl shifts are classified as allowed 5-*exo*-trig in Baldwin's Rules (which may not apply to a sulfur nucleophile).^23^ But for both O and S nucleophiles Baldwin's Rules offer no prohibition of either orientation of the C=O. The difference between *cis* and *trans* must be sought elsewhere.

**Scheme 2:**
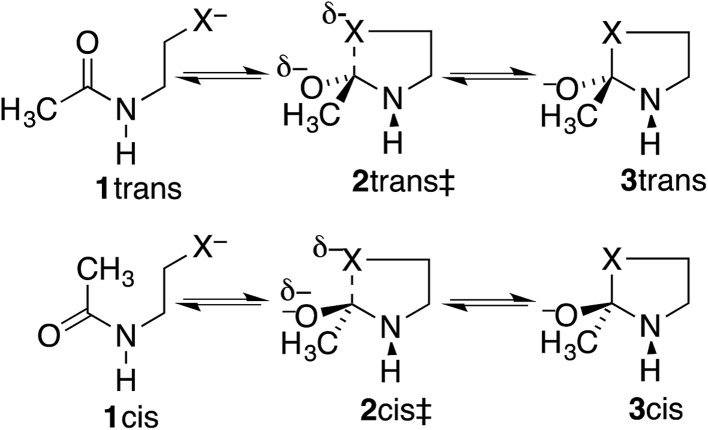
Cyclization of *trans* and *cis N*-CH_2_CH_2_X^–^-substituted acetamides (X = O or S).

Our proposal is that the *trans* amide reacts more slowly because its geometry restricts the nucleophilic O or S from approaching the C=O from the preferred direction. To test this proposal, we have calculated structures and energies for the intramolecular reactions of *cis* and *trans* acetamides **1** with O, S, and Se anions (simplified from OH, SH, and SeH nucleophiles activated through general-base catalysis by an appropriate amino-acid residue), *via* transition states **2**, leading to tetrahedral intermediates **3** (Scheme 2). Note that these amides are designated as the familiar *cis* and *trans*, rather than the *E* and *Z* recommended by IUPAC. Also, it may be noted that the two intermediates are of opposite configurations at the C undergoing addition, but the same configuration at NH, because the NH must remain either *trans* or *cis* to O. We claim that the calculated structures and energies provide a persuasive explanation for the geometric constraints required for the N-to-S or N-to-O acyl shift in cysteine and serine peptides.

## Methodology

DFT(B3LYP)/6-311++G(d,p) calculations were performed with Gaussian 09 software Revision C.01.^24^ Solvation by water was modeled with the polarized-continuum model.^25^ Transition states were found by the QST3 procedure and characterized by one negative (imaginary) frequency, while reactant amides and tetrahedral intermediates properly had no such frequencies.^26^

**Fig. 1:**
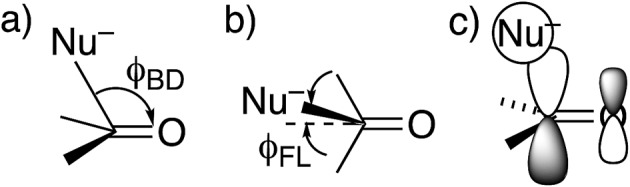
Approach of a nucleophile to a ketone or aldehyde carbonyl. (a) Side view of Bürgi–Dunitz angle. (b) Top view of Flippin–Lodge angle. (c) Overlap between the orbital on Nu^–^ and the π* MO of C=O.

The approach of a nucleophile to a carbonyl group is often specified by two angles. The more familiar is the Bürgi–Dunitz angle *φ*_BD_,^27^ between the C–Nu and C=O directions (Fig. 1a). The other is the lateral-displacement angle *φ*_FL_, between the C=O direction and the projection of the C–Nu direction onto the plane containing the C and the two attached groups (Fig. 1b). It is often called the Flippin–Lodge angle,^28^ which can describe the steric hindrance by bulky groups on the carbonyl. Both of these angles affect the overlap between the orbital on the nucleophile and the π* molecular orbital of the carbonyl (Fig. 1c).^29^ That overlap is maximized for *φ*_BD_ ∼107° and for *φ*_FL_ = 0, which thus specify the preferred direction of approach.

**Fig. 2:**
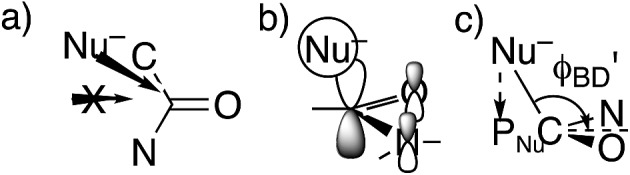
Approach of a nucleophile to an amide carbonyl. (a) Top view of approach *anti* to both O and N of an amide. (b) Overlap between the orbital on Nu^–^ and the amide π* MO. (c) Side view of modified Bürgi–Dunitz angle.

However, these angles are not appropriate for specifying the preferred direction of approach to an amide. Whereas the nucleophile approaches *anti* to the carbonyl O of an aldehyde or ketone, for amides the preferred approach is *anti* to both O and N, as suggested by the arrow in Fig. 2a and as has been rationalized in terms of the overlap between the orbital on the nucleophile and the π* molecular orbital of the amide (Fig. 2b).^30^ Indeed, according to the calculated transition state for OH^–^ addition to *trans-N*-methylacetamide or for HS^–^ addition to *trans-N*-methylacetamide ·HCl, the preferred *φ*_FL_ is not 0° but 52° or 61°, respectively. The Bürgi–Dunitz angle must then be modified as the complement of the Nu–C–P_Nu_ angle (Fig. 2c, where P_Nu_ is the projection of the nucleophile onto the NCO plane), which we designate as *φ*′_BD_.

The modified Bürgi–Dunitz angle *φ*′_BD_ was calculated as follows: first **X**_normal_, the normal to the OCN plane containing C, O, and N, was calculated as (**X**_O_ – **X**_C_) × (**X**_N_ – **X**_C_), the cross product between the C–O and C–N vectors. Next **P**_Nu_, the projection of **X**_Nu_ onto the NCO plane, was calculated as **X**_Nu_ + **X**_normal_(**X**_C_·**X**_normal_ – **X**_Nu_ × **X**_normal_)/**X**_normal_ × **X**_normal_. Finally, cos(180° – *φ*′_BD_) was evaluated as the normalized dot product (**X**_Nu_ – **X**_C_)(**P**_Nu_ – **X**_C_)/|**X**_Nu_ – **X**_C_||**P**_Nu_ – **X**_C_|.

## Results

Table 1 lists calculated energies of *cis* and *trans* stereoisomers of extended-chain amide **1**, tetrahedral intermediate **3**, and transition state **2** connecting them, for X = O, S, and Se, along with the activation energies *E*_a_ = *E*(**2**) – *E*(**1**). For X = S and Se either HF or HCl, respectively, was coordinated to the carbonyl oxygen in order to converge addition to the amide, which otherwise is thermodynamically unfavorable because RS^–^ and RSe^–^ are such stable anions and because C–S and C–Se bonds are weak. Besides, the coordinated acid can mimic the “oxyanion hole”, which stabilizes the tetrahedral intermediate in some enzyme-catalyzed reactions.

**Table 1:** Calculated relative energies (kcal mol^–1^) for N-to-X (X = O, S, Se) acyl transfer in **1**

	X = O	X = S^*a*^	X = Se^*b*^
*E*(**1***cis*)	1.7	2.0	1.9
*E*(**1***trans*)	≡0.0	≡0.0	≡0.0
*E*(**3***cis*)	10.1	14.1	4.6
*E*(**3***trans*)	12.6	14.5	4.2
*E*(**2‡***cis*)	10.5	14.6	6.7
*E*(**2‡***trans*)	13.3	17.9	7.4
*E*_a_(*cis*)	8.8	13.6	4.8
*E*_a_(*trans*)	13.3	17.9	7.4

^*a*^HF.

^*b*^HCl.

Because the coordination to HF or to HCl, the use of anionic nucleophiles, and the use of PCM are all devices to facilitate the calculations, the absolute energies in Table 1 cannot be compared to experimental energies. Nevertheless, the *trans* amides are a reasonable 2 kcal mol^–1^ more stable than the *cis*, and the open-chain amides **1** are calculated to be more stable than the high-energy tetrahedral intermediates **3**. Moreover, nearly the same results for X = O are obtained with the M06-2X method, which accounts for dispersion,^31^ as documented in Table S1,† and also with B3LYP/6-311++G(d,p) free energies, which include vibrational frequencies and zero-point energies, as documented in Table S2.†

**Fig. 3:**
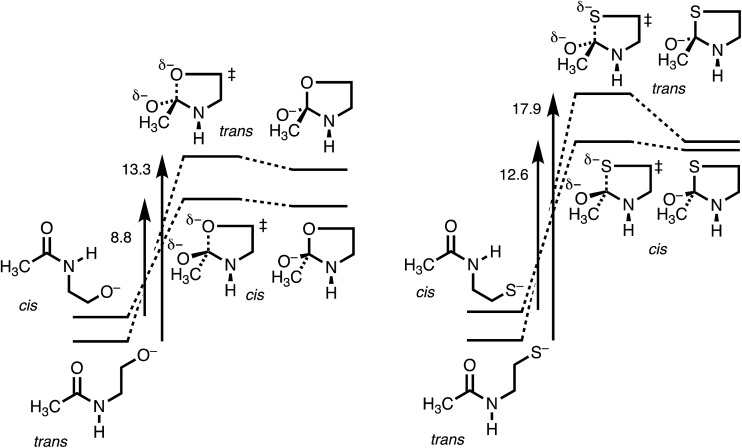
Energy diagram (kcal mol^–1^) for N-to-X (X = O, S) acyl transfer in **1**.

Fig. 3 makes the energies in Table 1 graphic. The key result is the lower energy of the *cis* transition state for both X = O (without HF) and X = S (with HF), by ∼2.5 kcal mol^–1^. The differences in activation energies are slightly larger, 4–5 kcal mol^–1^. These differences thus reproduce the faster cyclization seen for *cis* amides. However, the faster cyclization is not merely because of the destabilization of a *cis* amide, but because of the lower energy of the *cis* transition state, consistent with the Curtin–Hammett Principle. The case of X = Se, omitted from Fig. 3, is discussed below.

The details of the transition-state structures clarify these relative reactivities. Table 2 presents key distances and angles, and Fig. S1† shows views of these structures. The C–X bond that is being formed is properly longer in the transition state than when the bond is fully formed in intermediate **3**. The lengthening is greater for **2***trans*, especially for X = O (as seen in the MOs in Fig. S2†), and this may reflect a better overlap for the *cis* stereoisomer, but it is not conclusive. The XCO angles in intermediates **3** are close to tetrahedral, as expected. The XCO angles in the transition states are also near tetrahedral, and deviate from the preferred Bürgi–Dunitz angle of 107°, but not by much.

**Table 2:** Calculated C–X distances (Å), Bürgi–Dunitz (XCO) angles (°), modified Bürgi–Dunitz angles *φ*′_BD_ (°) in transition states for acyl transfer

	*d*_C–O_	*d*_C–S_	*d*_C–Se_	*θ*_OCO_	*θ*_SCO_ ^*a*^	*θ*_SeCO_ ^*b*^	*φ*′_BD_(O)	*φ*′_BD_(S)^*a*^	*φ*′_BD_(Se)^*b*^
NMA^*c*^	1.92	2.36^*b*^		106.9	103.4^*b*^		118.6	118.3^*b*^	
**2‡** *cis*	1.84	2.27^*a*^	2.69	110.5	110.5^*a*^	109.6	117.9	117.5^*a*^	112.5
**2‡** *trans*	1.86	2.30^*a*^	2.70	107.4	105.7^*a*^	100.3	113.4	111.2^*a*^	101.7

^*a*^HF.

^*b*^HCl.

^*c*^ *N*-Methylacetamide.

The most informative parameter is the modified Bürgi–Dunitz angle *φ*′_BD_ (Fig. 2c). The values should be compared with the 118.6° or 118.3° calculated for unconstrained addition of OH^–^ or HS^–^ to *N*-methylacetamide (NMA) or *N*-methylacetamide HCl. The smaller angles in the cyclic transition states and especially in the *trans* transition states represent a greater displacement of the nucleophile from the π* MO of the amide group (Fig. 2b), and a correspondingly greater loss of overlap, which raises the energy of the cyclic transition states, and especially the *trans*. However, the displacements are small and cannot readily be detected in Fig. S1.†

## Discussion

The data in Table 1 show that the DFT calculations reproduce the greater reactivity of the *cis* amides. The data in Table 2 show that the difference between *cis* and *trans* transition states lies in the ability of the nucleophile to approach the carbonyl group from the preferred direction. This is not simply a steric effect. Although the greater reactivity of a *cis* peptide (or of an *N*-alkylated peptide) is due to the greater proportion of a stereoisomer that cyclizes more rapidly, the reason for the faster cyclization is the ease of nucleophilic approach. Moreover, the approach is not simply *anti* to the carbonyl oxygen, but *anti* to both O and N.

Approach control has previously been recognized as arising from steric repulsions in the transition state, as in hydride reduction of cyclohexanones.^32^ Here it is a stereoelectronic effect, 33 arising instead from orbital overlaps in the transition state, which are more favorable for one direction of approach over the other. The more difficult approach of the nucleophile in the *trans* amide is a consequence of a greater restriction on the ability of the nucleophile to reach the carbonyl carbon, as manifested by *φ*′_BD_ < optimum. This is a constraint of the five-membered ring being formed. It is an unusual example of angle strain that differs between *cis* and *trans*, even though they are both 5-*exo*-trig.

This difference in angle strain is recognizable even with a simple molecular-model kit. Therefore we expect that the order of relative energies in Table 1 is not an artifact of our particular computational model but will be obtained by any such calculation.

The N-to-Se acyl shift in a selenocysteine residue provides an instructive contrast. According to the calculated energies in Table 1, the activation energy for the *trans* is substantially higher than that for the *cis*. This is simply because of the ground-state steric destabilization of the *cis*, as originally proposed to explain its greater reactivity. However, the data also show that the transition-state energies for *cis* and *trans* differ by less than 1 kcal mol^–1^, much less than the 4–5 kcal mol^–1^ for N-to-O and N-to-S shifts. Those shifts of a *trans* amide are retarded by the inability of the nucleophile to reach the carbonyl carbon. However, because the C–Se bond is longer, the nucleophilic selenium has less difficulty in reaching the carbonyl carbon. However, according to the values in Table 2, the modified Bürgi–Dunitz angle *φ*′_BD_ is again significantly smaller in the *trans* transition state, just as for X = O or S, so that this parameter does not reflect the slight difference in transition-state energies. This may be a consequence of C–Se–C angles in both transition states that are constrained near 80°, thereby distorting the five-membered ring.

As a corollary, there may be no strong constraint on the approach of the selenium to the carbonyl carbon. Although a bis(selenylethyl) peptide readily undergoes a N-to-Se acyl shift,^34^ the disubstitution may not be necessary. We suggest that a *trans* mono(selenylethyl) peptide might suffice, although it would be retarded by the lack of the ground-state steric destabilization of the *cis* isomer.

**Scheme 3:**
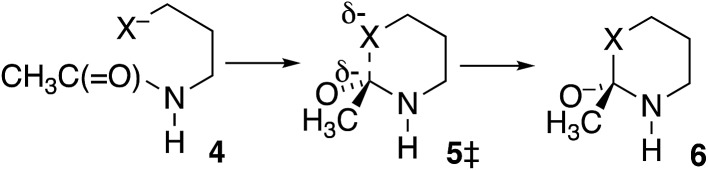
Cyclization of homologous *N*-CH_2_CH_2_CH_2_X^–^-substituted acetamides (X = O or S).

In further contrast, according to the data in Table 3, the transition-state energies for cyclization of homolog **4** (X = O) or **4** HF (X = S) are nearly equal for *cis* and *trans* amides Scheme 3). Moreover, for X = O the preferred *φ*_FL_ is calculated to be 55° for *cis* and 50° for *trans*, not far from the 52° for OH^–^ addition to *N*-methylacetamide. With a six-membered ring there is little restriction on the ability of the nucleophile to reach the carbonyl carbon. Indeed, the N-to-S acyl shift in a homocysteine residue is facile, without any necessity for *N*-alkylation or population of the *cis* amide.^35^ Likewise, there is no such necessity with class 2 and 3 inteins, where a distant nucleophile adds to the carbonyl and forms a macrocycle.^36^

**Table 3:** Calculated energies (kcal mol^–1^, relative to **4***trans*) of transition states **5‡** for cyclization of **4**

	X = O	X = S^*a*^
*E*(**5‡***cis*)	11.39	15.84
*E*(**5‡***trans*)	11.44	15.67

^*a*^HF.

**Scheme 4:**
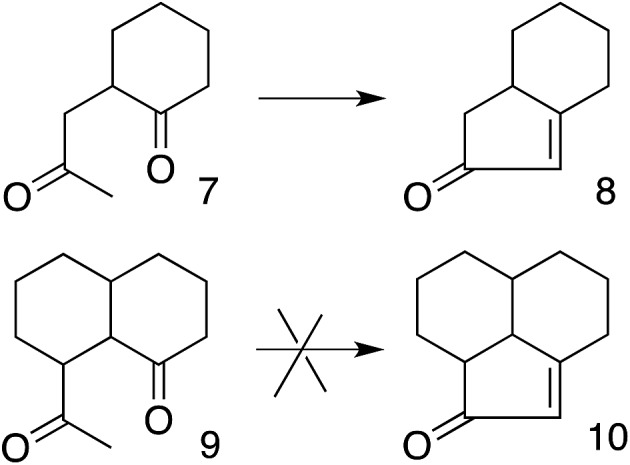
Constraint on aldol condensation.

These results also have implications for aldol condensation (Scheme 4). Although many conversions of enolate **7** and its derivatives to the corresponding **8** are known,^37^ we can find no report of the conversion of any derivative of **9** to the corresponding **10**. This could be due simply to a lack of demand for **10**, but it can also be explained by the difficulty for the enolate carbon of **9** to reach the carbonyl carbon, whereas **7** can twist to allow its enolate carbon to achieve the preferred approach to the carbonyl, as described by the Bürgi–Dunitz and Flippin–Lodge angles.

## Conclusions

DFT calculations can reproduce the greater reactivity of a *cis* acylcysteine or acylserine toward N-to-S or N-to-O acyl shift. The reactivity difference between *cis* and *trans* can be attributed to the ease of approach to the carbonyl carbon by the nucleophile, not to ground-state destabilization. This represents an extension of Baldwin's rules to two distinguishable cases of 5-*exo*-trig ring closures. Moreover, there is no large reactivity difference in an N-to-Se acyl shift or in 6-*exo*-trig ring closures. We thus have provided a better understanding of the geometric constraints required for the N-to-S or N-to-O acyl shift in cysteine and serine peptides. We expect that this information will enable better design of peptides, especially ones containing homocysteine, that generate thioesters useful for chemical ligation techniques.

## Conflicts of interest

There are no conflicts of interest to declare.

## Supplementary Material

Supplementary information
