# Supplementary material for: Approach control. Stereoelectronic origin of geometric constraints on N-to-S and N-to-O acyl shifts in peptides
Source: Chem Sci. 2018 Jan 8;9(7):1789–94. doi: 10.1039/c7sc04046f (PMC5892126; doi:10.1039/c7sc04046f)
Supplement: Supplementary file 1 [file SC-009-C7SC04046F-s001.pdf]

Electronic Supplementary Material (ESI) for Chemical Science.
This journal is © The Royal Society of Chemistry 2018

Supporting Information for

Approach Control. Stereoelectronic Origin of geometric constraints on N-to-S and N-to-O acyl shifts in peptides

Neal K. Devaraj* and Charles L. Perrin*

Dept. of Chemistry & Biochemistry, Univ. Calif. San Diego

La Jolla, CA 92093-0358 USA

Calculated energies. B3LYP/6-311++G(d,p) DFT energies are listed in Table S1.

Table S1 DFT Energies (A.U.).

Structure	$-E_{cis}$	$-E_{trans}$
**NMA**+OH^-		-324.556613193
**NMA·HCl**+HS^-		-1108.43106504
**1** (X=O)	-362.678464086	-362.681093900
**3** (X=O)	-362.664944698	-362.661047892
**2**‡ (X=O)	-362.664413383	-362.659941031
**1**·HF (X=S)	-786.195116739	-786.198258999
**3**·HF (X=S)	-786.175867912	-786.175158328
**2**‡·HF (X=S)	-786.175068460	-786.169713427
**1**·HCl (X=Se)	-3149.88875116	-3149.89174427
**3**·HCl (X=Se)	-3149.88449239	-3149.88510409
**2**‡·HCl (X=Se)	-3149.88104526	-3149.87996467
**5**‡ (X=O)	-401.984101279	-401.984031018
**5**‡·HF (X=S)	-825.495035167	-825.495302819

Calculated coordinates. B3LYP/6-311++G(d,p) Cartesian coordinates are listed in Tables S2-S13.

Table S2. Optimized coordinates (Å) for **1** (X=O), $CH_3CONHCH_2CH_2O^-$.

Atom	x_{cis}	y_{cis}	z_{cis}	x_{trans}	y_{trans}	z_{trans}
C	0.888736	-0.207638	0.684856	-0.948264	-0.053758	0.67791
H	1.314236	-0.823456	1.483022	-0.85302	1.012804	0.884521

H	0.764678	0.798978	1.087999	-1.328031	-0.543693	1.580389
C	1.885909	-0.167741	-0.504337	-1.960459	-0.27108	-0.479601
H	1.37876	0.42942	-1.303728	-1.933942	-1.3684	-0.703066
H	1.906833	-1.209501	-0.914179	-1.493822	0.202868	-1.378944
N	-0.427464	-0.759131	0.347031	0.389833	-0.573925	0.395162
H	-0.49378	-1.764897	0.255591	0.506702	-1.576026	0.390402
O	3.114401	0.311061	-0.150332	-3.209587	0.199708	-0.189697
C	-1.544109	-0.097206	-0.030311	1.449069	0.177595	0.022253
O	-2.561311	-0.707232	-0.393038	1.41287	1.412461	-0.048326
C	-1.520761	1.417325	0.038125	2.723345	-0.580451	-0.299869
H	-1.430057	1.753084	1.074902	2.629781	-1.66072	-0.180189
H	-0.679562	1.833184	-0.519802	3.012671	-0.361697	-1.330306
H	-2.452232	1.798038	-0.376061	3.522422	-0.218843	0.351084

Table S3. Optimized coordinates (Å) for **3** (X=O), *cyclo*-$NHCH_2CH_2OC(CH_3)O^-$.

Atom	x_{cis}	y_{cis}	z_{cis}	x_{trans}	y_{trans}	z_{trans}
C	1.481263	-0.814501	-0.134296	1.548063	-0.739584	-0.134356
H	1.466016	-1.144543	-1.178522	2.31081	-1.107851	-0.823576
H	2.292359	-1.340975	0.374567	1.686728	-1.235314	0.829624
C	1.602749	0.715863	-0.061953	1.569503	0.793155	0.02983
H	2.255325	1.120377	-0.846262	1.928146	1.269165	-0.89751
H	2.024079	1.014431	0.913781	2.230192	1.111405	0.84674
N	0.189331	-1.101978	0.493531	0.19746	-1.025638	-0.632974
H	0.292841	-1.062213	1.505273	0.166504	-0.727786	-1.606539
O	0.284216	1.190187	-0.222643	0.228055	1.16507	0.289953
C	-0.753578	0.001875	0.171293	-0.70354	-0.126021	0.153225
O	-1.526695	0.346461	1.150769	-1.032975	-0.574433	1.325841
C	-1.459286	-0.266079	-1.169409	-1.871542	0.358887	-0.725111
H	-2.206942	-1.052587	-1.027536	-2.549818	-0.47739	-0.919166
H	-1.968802	0.642641	-1.498753	-2.427242	1.12806	-0.18445
H	-0.767255	-0.57941	-1.956078	-1.543084	0.775463	-1.682195

Table S4. Optimized coordinates (Å) for **2‡** (X=O), *cyclo*-$NHCH_2CH_2O\cdot\cdot C(CH_3)O^{-‡}$.

Atom	x_{cis}	y_{cis}	z_{cis}	x_{trans}	y_{trans}	z_{trans}
C	-1.417663	0.808617	0.34088	1.485892	-0.797564	-0.164163
H	-2.191726	1.510665	0.019908	2.231642	-1.217682	-0.843661
H	-1.364313	0.843622	1.433625	1.545621	-1.330163	0.787927
C	-1.663536	-0.641457	-0.133584	1.640033	0.729973	0.048262
H	-2.114639	-0.615472	-1.146174	2.015403	1.179584	-0.893892
H	-2.390163	-1.143019	0.527095	2.40061	0.931011	0.821543
N	-0.121581	1.187744	-0.223784	0.134167	-0.964544	-0.71062
H	-0.228028	1.414565	-1.209726	0.128664	-0.565809	-1.646404
O	-0.420925	-1.269728	-0.121464	0.383919	1.221777	0.398823
C	0.823261	0.088059	-0.168533	-0.78027	-0.200894	0.125268
O	1.595259	-0.051107	-1.16619	-1.074656	-0.672649	1.262066
C	1.42126	-0.125825	1.222107	-1.88242	0.497068	-0.67129
H	1.849558	-1.127608	1.280627	-2.645259	-0.239663	-0.946764
H	2.224456	0.604854	1.371889	-2.354533	1.251054	-0.040074
H	0.691322	-0.011508	2.025256	-1.514831	0.978947	-1.579908

Table S5. Optimized coordinates (Å) for **1**·HF (X=S), $CH_3CONHCH_2CH_2S^{-}\cdot HF$.

Atom	x_{cis}	y_{cis}	z_{cis}	x_{trans}	y_{trans}	z_{trans}
C	1.148057	-0.386725	0.660028	1.057603	-0.476639	0.649829
H	1.605744	-1.075865	1.372732	1.58629	-1.094914	1.378151
H	1.06747	0.579782	1.156017	0.773308	0.455019	1.136928
C	2.025697	-0.271805	-0.590209	1.960744	-0.194701	-0.554977
H	1.54705	0.410738	-1.298774	1.409667	0.421494	-1.270892
H	2.080783	-1.251824	-1.073564	2.193676	-1.140899	-1.052412
N	-0.203837	-0.889692	0.370866	-0.177768	-1.18221	0.280801
H	-0.302438	-1.885607	0.213991	-0.119284	-2.177738	0.122866
S	3.727931	0.326861	-0.189	3.528435	0.65568	-0.065903
C	-1.326362	-0.186061	0.196992	-1.357841	-0.593429	0.071274

O	-2.390028	-0.783405	-0.101487	-1.504471	0.642558	0.219201
F	-4.547555	0.305656	-0.452032	-3.630975	1.812145	-0.069302
H	-3.659986	-0.108386	-0.303486	-2.784123	1.310324	0.038798
C	-1.290629	1.309985	0.38651	-2.5121	-1.471619	-0.341994
H	-1.095485	1.552529	1.434338	-2.896884	-1.124542	-1.303416
H	-0.503862	1.768194	-0.214844	-2.2342	-2.521663	-0.427855
H	-2.251667	1.732479	0.101785	-3.314938	-1.37393	0.39198

Table S6. Optimized coordinates (Å) for **3**·HF (X=S), *cyclo*-$NHCH_2CH_2SC(CH_3)O^-$·HF.

Atom	x_{cis}	y_{cis}	z_{cis}	x_{trans}	y_{trans}	z_{trans}
C	-2.111726	0.793947	-0.049695	1.893882	0.506301	-0.907257
H	-2.90701	1.299207	-0.601919	2.828216	1.056039	-1.037528
H	-2.220587	1.05601	1.005634	1.487753	0.281809	-1.894701
C	-2.192901	-0.737912	-0.214454	2.1158	-0.801566	-0.117293
H	-2.426278	-1.007081	-1.24675	2.855685	-0.661104	0.673238
H	-2.948608	-1.171628	0.441843	2.444922	-1.612666	-0.76782
N	-0.817406	1.264983	-0.533935	0.930408	1.340856	-0.186071
S	-0.528509	-1.357862	0.240229	0.487101	-1.21333	0.63124
C	0.284771	0.496161	-0.017081	-0.249973	0.581623	0.132836
O	1.28378	0.475467	-0.93482	-1.031974	0.453085	-0.984287
F	3.356045	-0.506004	-0.197262	-3.12235	-0.745033	-0.592729
H	2.181321	0.048099	-0.567984	-1.91265	-0.067411	-0.782663
C	0.729816	0.947647	1.371317	-0.99785	1.143024	1.336782
H	1.458972	0.248326	1.781761	-1.384467	2.134509	1.081796
H	1.197249	1.933715	1.282761	-1.843793	0.500767	1.582958
H	-0.112768	1.014876	2.059908	-0.350355	1.223896	2.21151
H	-0.778707	1.200623	-1.547966	1.35401	1.67577	0.67431

Table S7. Optimized coordinates (Å) for **2**‡·HF (X=S), *cyclo*-$NHCH_2CH_2SC(CH_3)O^-$·HF‡.

Atom	x_{cis}	y_{cis}	z_{cis}	x_{trans}	y_{trans}	z_{trans}
C	2.00713	-0.956031	0.011314	-1.871204	-0.467896	-0.946552

H	2.753275	-1.58714	-0.476916	-2.805946	-1.007629	-1.110466
H	2.049379	-1.1608	1.083113	-1.447598	-0.212621	-1.918599
C	2.280998	0.542892	-0.244276	-2.102425	0.81724	-0.117583
H	2.549183	0.704128	-1.291481	-2.877527	0.663032	0.635558
H	3.108384	0.88831	0.378559	-2.392381	1.654015	-0.753885
N	0.687382	-1.312309	-0.495983	-0.924777	-1.331833	-0.240535
H	0.670801	-1.307906	-1.511956	-1.360847	-1.698994	0.599858
S	0.740541	1.444899	0.164901	-0.504508	1.173462	0.719388
C	-0.380229	-0.516807	-0.028699	0.256843	-0.594532	0.119532
O	-1.35279	-0.408281	-0.909936	1.017653	-0.387151	-0.998181
F	-3.39439	0.492603	-0.192445	3.103639	0.806101	-0.592235
H	-2.332803	0.028739	-0.521054	1.898974	0.13007	-0.784552
C	-0.797836	-0.76344	1.411777	1.019512	-1.238958	1.292493
H	-1.452925	0.035537	1.756502	0.56225	-2.195127	1.559937
H	-1.351484	-1.709517	1.445873	2.0504	-1.430856	0.9944
H	0.057315	-0.830432	2.081621	1.027914	-0.607279	2.179507

Table S8. Optimized coordinates (Å) for **1**·HCl (X=Se), $CH_3CONHCH_2CH_2Se^{-}$·HCl.

Atom	x_{cis}	y_{cis}	z_{cis}	x_{trans}	y_{trans}	z_{trans}
C	-0.852952	0.407838	0.664255	0.773216	0.750019	-0.633676
H	-0.741682	-0.610763	1.029016	0.463544	-0.148808	-1.162476
H	-1.265359	1.007293	1.477333	1.319748	1.388211	-1.328445
C	-1.766979	0.455814	-0.555975	1.635630	0.406503	0.578179
H	-1.339704	-0.140887	-1.363135	1.076050	-0.236293	1.258917
H	-1.863646	1.484583	-0.906992	1.904768	1.318749	1.112934
C	1.619820	0.299059	0.266892	-1.640191	0.978169	-0.077873
O	2.667331	1.006412	-0.024395	-1.788479	-0.290599	-0.281017
H	3.541133	0.471665	-0.112044	-2.751518	-0.634077	-0.147008
N	0.491379	0.952877	0.370452	-0.450533	1.493331	-0.252908
Se	-3.587554	-0.247050	-0.119018	3.313080	-0.533911	0.028379
H	0.549028	1.951736	0.196450	-0.351882	2.483323	-0.068697

Cl	5.225292	-0.340043	-0.324816	-4.438375	-1.415939	0.049413
C	1.713868	-1.175448	0.489264	-2.789538	1.835369	0.337677
H	1.542721	-1.395883	1.546170	-3.205005	1.454125	1.272579
H	0.959351	-1.702795	-0.094802	-2.487074	2.872300	0.469501
H	2.704204	-1.529522	0.211853	-3.574109	1.777527	-0.419564

Table S9. Optimized coordinates (Å) for **3**·HCl (X=Se), *cyclo*-$NHCH_2CH_2SeC(CH_3)O^{-}$·HCl.

Atom	x_{cis}	y_{cis}	z_{cis}	x_{trans}	y_{trans}	z_{trans}
C	2.443154	1.063370	-0.023794	-2.125806	0.871872	-1.061511
H	3.251378	1.608013	-0.517307	-3.071118	1.394795	-1.223234
H	2.540039	1.231420	1.050985	-1.580254	0.858883	-2.006148
C	2.528736	-0.446277	-0.323395	-2.368993	-0.570103	-0.573041
H	3.279248	-0.937734	0.294932	-2.580432	-1.247501	-1.399691
H	2.747412	-0.632932	-1.374873	-3.182878	-0.616769	0.150665
C	0.031663	0.892015	0.003814	-0.119987	0.944783	0.253947
O	-0.979504	1.023640	-0.924028	0.781144	1.120088	-0.790735
H	-1.820827	0.628297	-0.590717	1.617922	0.633377	-0.608606
N	1.167499	1.607043	-0.481984	-1.333672	1.613557	-0.078121
Se	0.736560	-1.163446	0.107676	-0.688403	-1.120389	0.322634
H	1.128980	1.611820	-1.497897	-1.878870	1.771536	0.763132
Cl	-3.687649	-0.251490	-0.115087	3.553903	-0.294084	-0.406390
C	-0.389546	1.280089	1.413961	0.444135	1.359467	1.603501
H	-0.764434	2.308929	1.394260	1.344427	0.787456	1.830967
H	-1.182691	0.621285	1.769745	0.712120	2.420140	1.564968
H	0.447386	1.219784	2.108967	-0.281090	1.199050	2.402376

Table S10. Optimized coordinates (Å) for **2**‡·HCl (X=Se), *cyclo*-$NHCH_2CH_2SeC(CH_3)O^{-}$ ·$HCl^{‡}$.

Atom	x_{cis}	y_{cis}	z_{cis}	x_{trans}	y_{trans}	z_{trans}
C	-2.060096	1.523941	-0.106567	-1.782751	1.346008	-0.973588
H	-2.707140	2.320083	0.268077	-2.540553	2.113103	-1.145670

H	-2.017125	1.613851	-1.191693	-1.181159	1.248654	-1.875645
C	-2.598820	0.135402	0.295790	-2.436642	-0.003907	-0.599262
H	-3.492300	-0.086926	-0.288397	-2.847626	-0.462412	-1.499169
H	-2.866738	0.128541	1.353518	-3.251925	0.159214	0.107250
C	0.307787	0.971526	0.012143	0.236868	1.103800	0.319273
O	1.264377	0.832440	0.916294	1.008666	0.974069	-0.747183
H	2.099236	0.410185	0.552221	1.846994	0.456636	-0.558653
N	-0.726302	1.734748	0.445266	-0.910123	1.790159	0.114937
Se	-1.214873	-1.242749	-0.037761	-1.111578	-1.231737	0.225680
H	-0.719230	1.843665	1.454756	-1.413197	1.971544	0.975208
Cl	3.849656	-0.430429	0.088199	3.589252	-0.520279	-0.416507
C	0.699500	0.967843	-1.439579	0.872005	1.131610	1.678172
H	1.301020	1.868985	-1.618431	1.576291	0.308554	1.782838
H	1.307417	0.094960	-1.667119	1.429391	2.070444	1.781480
H	-0.164733	0.982374	-2.096363	0.122797	1.069319	2.465176

Table S11. Optimized coordinates (Å) for **5**‡ (X=O), *cyclo*-$NHCH_2CH_2CH_2O{\cdot}{\cdot}C(CH_3)O^{-\ddagger}$.

Atom	x_{cis}	y_{cis}	z_{cis}	x_{trans}	y_{trans}	z_{trans}
C	-1.063948	1.294747	0.311564	1.155299	-1.30357	0.040205
H	-0.962918	1.244895	1.400697	1.110044	-1.476587	1.118175
H	-1.496578	2.274822	0.088221	1.609016	-2.191073	-0.412321
C	-1.357328	-1.204141	0.084173	1.271382	1.231344	0.210088
H	-2.037655	-1.988747	-0.296178	1.909908	2.103308	-0.023758
H	-1.324405	-1.355718	1.186468	1.204945	1.194331	1.317718
N	0.28025	1.264726	-0.283964	-0.229922	-1.197517	-0.447612
H	0.244088	1.475683	-1.276481	-0.222124	-1.035872	-1.449393
O	-0.098073	-1.325757	-0.500098	0.014083	1.376019	-0.368741
C	1.114469	0.11449	-0.123748	-1.01117	-0.175745	0.190805
O	2.032072	-0.027853	-0.977846	-1.054556	-0.160161	1.448617
C	-1.987413	0.173462	-0.172643	1.989033	-0.050862	-0.259005
H	-2.955746	0.251004	0.339298	2.971211	-0.138249	0.224812

H	-2.173283	0.293955	-1.248001	2.164738	0.018484	-1.341314
C	1.415637	-0.248156	1.327485	-2.254256	0.190356	-0.608744
H	1.935777	-1.20544	1.348134	-2.999347	-0.604557	-0.49172
H	2.077679	0.518832	1.746615	-2.673028	1.116932	-0.215976
H	0.530796	-0.315902	1.961542	-2.043858	0.319897	-1.672033

Table S12. Optimized coordinates (Å) for **5**‡·HF (X=S), *cyclo*-$NHCH_2CH_2CH_2S\cdot\cdot C(CH_3)O^-$ ·HF‡.

Atom	x_{cis}	y_{cis}	z_{cis}	x_{trans}	y_{trans}	z_{trans}
C	0.897445	-1.428664	0.983019	-1.202559	-1.654898	0.300354
H	1.291138	-0.932705	1.873546	-1.558693	-2.484197	0.916986
H	0.797789	-2.486818	1.236128	-0.73112	-2.088148	-0.584932
C	2.197616	0.177949	-0.557936	-1.961314	0.418677	-0.977823
H	2.944205	0.200692	-1.355252	-2.814371	1.050046	-1.232129
H	2.639434	0.689231	0.304408	-1.501802	0.081946	-1.910273
N	-0.458244	-0.92901	0.726941	-0.154966	-0.957462	1.062968
C	-0.744151	0.417631	0.620805	0.520291	0.126424	0.369315
O	-1.937001	0.714301	0.157048	1.08472	-0.334526	-0.798726
C	1.878507	-1.27172	-0.185909	-2.38748	-0.774658	-0.122569
H	2.809735	-1.77646	0.101949	-3.092928	-1.388625	-0.695504
H	1.487293	-1.801158	-1.062041	-2.919231	-0.416745	0.766592
H	-0.966857	-1.468096	0.030536	-0.541588	-0.607941	1.934993
C	-0.256385	1.330701	1.720745	1.505987	0.806886	1.315322
H	-0.479807	2.361759	1.455683	1.005508	1.150482	2.22307
H	-0.800364	1.074618	2.638793	2.287769	0.095464	1.589276
H	0.810247	1.238342	1.912337	1.972578	1.663911	0.826543
S	0.717332	1.128485	-1.132187	-0.765041	1.509681	-0.10154
H	-2.344537	-0.101684	-0.477259	2.127799	-0.40054	-0.766266
F	-2.755563	-1.068913	-1.152572	3.507132	-0.551855	-0.86692

Table S13. Optimized coordinates (Å) for $[CH_3CONHCH_3 + XH^-]^‡$ (X=O,S)

Atom	x_O	y_O	z_O	x_S[a]	y_S[a]	z_S[a]
C	1.648115	-0.749615	-0.467248	0.095155	-0.068756	1.824260
H	1.699686	-0.506565	-1.530928	0.804959	0.513281	2.411853
H	1.676946	-1.839651	-0.358235	-0.069022	-1.032344	2.318298
H	2.517219	-0.324359	0.033432	-0.851618	0.464718	1.771995
C	0.372612	-0.224596	0.187765	0.633284	-0.332198	0.438222
N	-0.779947	-0.525799	-0.603677	1.901207	-0.833025	0.383031
C	-2.070315	-0.06849	-0.099401	2.525306	-1.189220	-0.886187
H	-2.119252	1.021737	0.031465	2.654070	-0.312189	-1.531898
H	-2.851661	-0.36898	-0.801214	3.502493	-1.623434	-0.678397
H	-2.277495	-0.534439	0.864155	1.916984	-1.925984	-1.408004
O	0.25535	-0.271519	1.444977	-0.183744	-0.901153	-0.457193
O/S	0.684296	1.607673	-0.275949	0.745292	1.916909	-0.273348
H	0.230088	2.020876	0.466357	1.039554	1.607615	-1.552750
H	-0.635544	-0.221057	-1.558219	2.526883	-0.410732	1.054195
H				-1.130154	-0.615746	-0.337491
Cl				-3.157634	-0.279664	-0.291511

[a]·HCl.

Table S14. Relative M06-2X/6-311++G(d,p) energies (kcal/mol) for N-to-O acyl transfer in **1 (X=O).**

E(**1cis**)	1.00
E(**1trans**)	≡0.00
E(**3cis**)	3.55
E(**3trans**)	5.25
E(**2‡cis**)	7.30
E(**2‡trans**)	9.92
E_a(**cis**)	6.31
E_a(**trans**)	9.92

Table S15. Relative B3LYP/6-311++G(d,p) free energies (kcal/mol) for N-to-O acyl transfer in **1 (X=O).**

G^o(**1cis**)	1.82
G^o(**1trans**)	≡0.00
G^o(**3cis**)	12.94
G^o(**3trans**)	14.95
G^o(**2‡cis**)	13.10
G^o(**2‡trans**)	15.47
G^o_a(**cis**)	11.28
G^o_a(**trans**)	15.47

Table S16. Calculation of modified Bürgi-Dunitz angle for NMA + $OH^–$ or NMA·HCl + $SH^–$.

	x	*y*	*z*		*x*	*y*	*z*	
C	0.373	-0.225	0.188		0.633	-0.332	0.438	
N	-0.780	-0.526	-0.604		1.901	-0.833	0.383	
O	0.255	-0.272	1.445		-0.184	-0.901	-0.457	
Nu	0.684	1.608	-0.276		0.745	1.917	-0.273	
				sum				*sum*
$\mathbf{X}_N$-$\mathbf{X}_C$	-1.153	-0.301	-0.791		1.268	-0.501	-0.055	
$\mathbf{X}_O$-$\mathbf{X}_C$	-0.117	-0.047	1.257		-0.817	-0.569	-0.895	
normal	-0.416	1.542	0.019		0.417	1.180	-1.131	
$\mathbf{X}_C$·normal	-0.155	-0.346	0.004	-0.498	0.264	-0.392	-0.495	-0.623
$\mathbf{X}_{Nu}$·normal	-0.285	2.479	-0.005	2.189	0.311	2.263	0.309	2.883
normal·normal	0.173	2.377	0.000	2.550	0.174	1.393	1.278	2.846
ratio	-1.053				-1.232			
$\mathbf{P}_{Nu}$	1.122	-0.017	-0.296		0.231	0.462	1.120	
$\mathbf{X}_C$-$\mathbf{P}_N$u	0.750	0.208	-0.483	0.839	-0.402	0.795	0.681	1.257
$\mathbf{X}_C$-$\mathbf{X}_{Nu}$	0.312	1.832	-0.464	3.669	0.112	2.249	-0.712	5.577
dotpdt				0.839				1.257
normalized				0.478				0.475
$\phi_{BD'}$				118.6				118.3

Table S17. Calculation of modified Bürgi-Dunitz angle for **2‡** (X=O).

	x_{cis}	y_{cis}	z_{cis}		x_{trans}	y_{trans}	z_{trans}	
N	0.122	-1.188	-0.224		0.134	-0.965	-0.711	

Nu	0.421	1.270	-0.121		0.384	1.222	0.399	
C	-0.823	-0.088	-0.169		-0.780	-0.201	0.125	
O	-1.595	0.051	-1.166		-1.075	-0.673	1.262	
				sum				*sum*
$\mathbf{X}_N$-$\mathbf{X}_C$	0.945	-1.100	-0.055		0.914	-0.764	-0.836	
$\mathbf{X}_O$-$\mathbf{X}_C$	-0.772	0.139	-0.998		-0.294	-0.472	1.137	
normal	1.105	0.985	-0.717	1.645	-1.262	-0.793	-0.656	1.629
$\mathbf{X}_C$·normal	-0.910	-0.087	0.121	-0.875	0.985	0.159	-0.082	1.062
$\mathbf{X}_{Nu}$·normal	0.465	1.251	0.087	1.803	-0.485	-0.969	-0.262	-1.716
normal·normal	1.221	0.971	0.515	2.706	1.594	0.630	0.431	2.654
ratio	-0.990				1.047			
$\mathbf{P}_{Nu}$	-0.673	0.294	0.589		-0.938	0.391	-0.288	
$\mathbf{X}_C$-$\mathbf{P}_N$u	0.151	0.383	0.757	0.742	-0.157	0.592	-0.413	0.546
$\mathbf{X}_C$-$\mathbf{X}_{Nu}$	1.244	1.358	0.047	3.394	1.164	1.423	0.274	3.454
dotpdt	0.187	0.519	0.036	0.742	-0.183	0.842	-0.113	0.546
normalized				0.468				0.398
$\phi_{BD'}$				117.9				113.4

Table S18. Calculation of modified Bürgi-Dunitz angle for **2**‡·HF (X=S).

	x_{cis}	y_{cis}	z_{cis}		x_{trans}	y_{trans}	z_{trans}	
N	0.687	-1.312	-0.496		0.779	1.408	-0.003	
Nu	0.741	1.445	0.165		0.729	-1.370	0.502	
C	-0.380	-0.517	-0.029		-0.373	0.627	0.181	
O	-1.353	-0.408	-0.910		-1.096	0.464	-0.903	
				sum				*sum*
$\mathbf{X}_N$-$\mathbf{X}_C$	1.068	-0.796	-0.467		1.153	0.781	-0.184	
$\mathbf{X}_O$-$\mathbf{X}_C$	-0.973	0.109	-0.881		-0.722	-0.163	-1.084	
normal	0.752	1.395	-0.658	1.716	-0.877	1.383	0.376	1.680
$\mathbf{X}_C$·normal	-0.286	-0.721	0.019	-0.988	0.328	0.867	0.068	1.262
$\mathbf{X}_{Nu}$·normal	0.557	2.016	-0.108	2.464	-0.639	-1.895	0.189	-2.345
normal·normal	0.565	1.947	0.433	2.945	0.769	1.912	0.141	2.823
ratio	-1.172				1.278			
$\mathbf{P}_{Nu}$	-0.141	-0.191	0.936		-0.392	0.397	0.983	
$\mathbf{X}_C$-$\mathbf{P}_N$u	0.239	0.326	0.965	1.094	-0.019	-0.230	0.802	0.696
$\mathbf{X}_C$-$\mathbf{X}_{Nu}$	1.121	1.962	0.194	5.142	1.102	-1.997	0.321	5.308
dotpdt	0.268	0.639	0.187	1.094	-0.021	0.459	0.257	0.696
normalized				0.461				0.362
$\phi_{BD'}$				117.5				111.2

Table S19. Calculation of modified Bürgi-Dunitz angle for $\mathbf{2}^{\ddagger}\cdot$HCl (X=Se).

	x_{cis}	y_{cis}	z_{cis}		x_{trans}	y_{trans}	z_{trans}	
C	0.308	0.972	0.012		0.237	1.104	0.319	
O	1.264	0.832	0.916		1.009	0.974	-0.747	
N	-0.726	1.735	0.445		-0.910	1.790	0.115	
Se=Nu	-1.215	-1.243	-0.038		-1.112	-1.232	0.226	
				sum				*sum*
$\mathbf{X}_N$-$\mathbf{X}_C$	-1.034	0.763	0.433		-1.147	0.686	-0.204	
$\mathbf{X}_O$-$\mathbf{X}_C$	0.957	-0.139	0.904		0.772	-0.130	-1.066	
normal	0.750	1.349	-0.586	1.651	-0.758	-1.381	-0.381	1.621
$\mathbf{X}_C\cdot$normal	0.231	1.311	-0.007	1.535	-0.180	-1.524	-0.122	-1.826
$\mathbf{X}_{Nu}\cdot$normal	-0.912	-1.677	0.022	-2.566	0.843	1.701	-0.086	2.458
normal·normal	0.563	1.821	0.344	2.727	0.575	1.907	0.145	2.627
ratio	1.504				-1.630			
$\mathbf{P}_{Nu}$	-0.087	0.786	-0.919		0.125	1.020	0.847	
$\mathbf{X}_C$-$\mathbf{P}_N$u	-0.394	-0.185	-0.931	1.058	-0.112	-0.084	0.527	0.298
$\mathbf{X}_C$-$\mathbf{X}_{Nu}$	-1.523	-2.214	-0.050	7.224	-1.348	-2.336	-0.094	7.282
dotpdt	0.601	0.410	0.046	1.058	0.151	0.196	-0.049	0.298
normalized				0.383				0.202
$\phi_{BD'}$				112.5				101.7

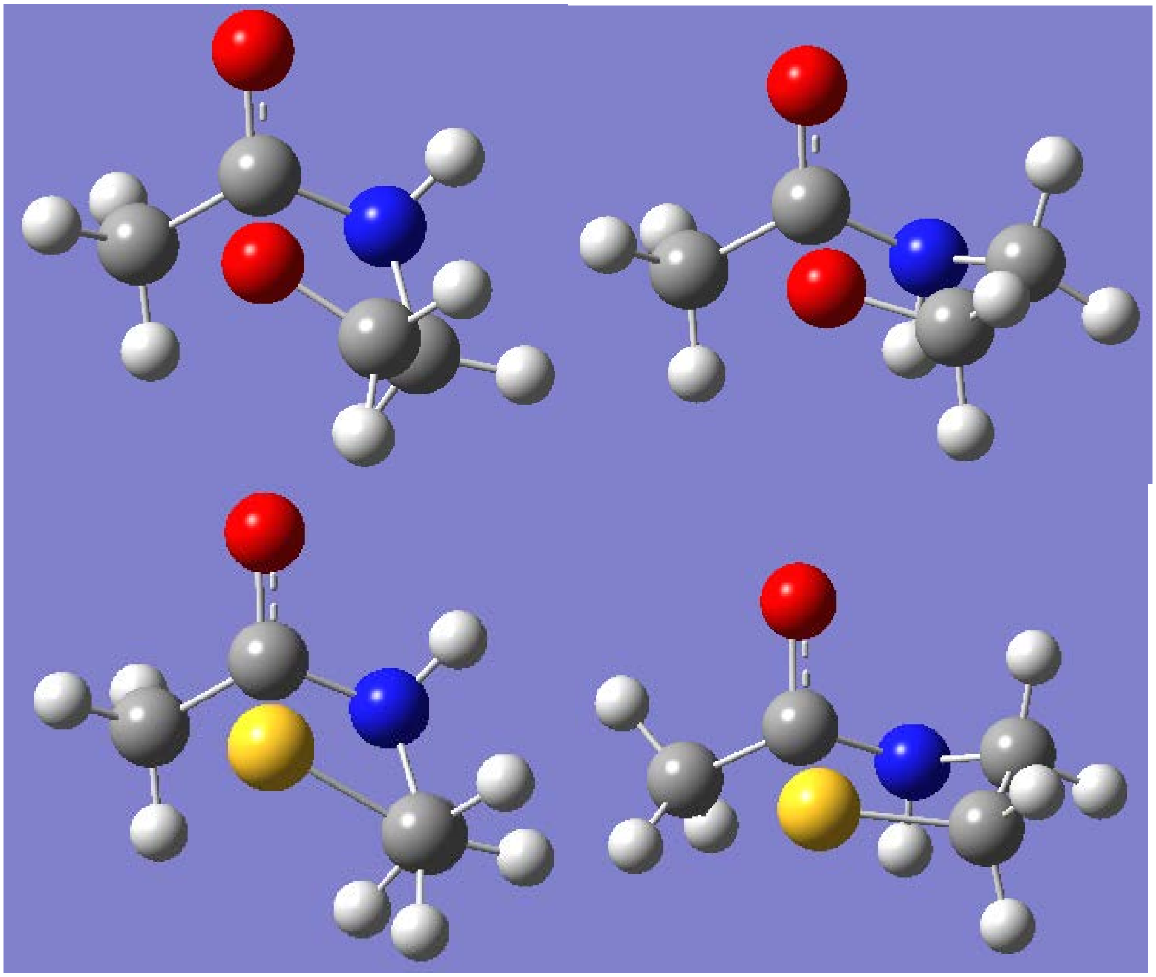

Figure S1. Calculated transition states for N-to-X acyl transfer, with HF omitted for clarity: **2cis‡** (X=O), **2trans‡** (X=O), **2cis‡** (X=S), **2trans‡** (X=S).

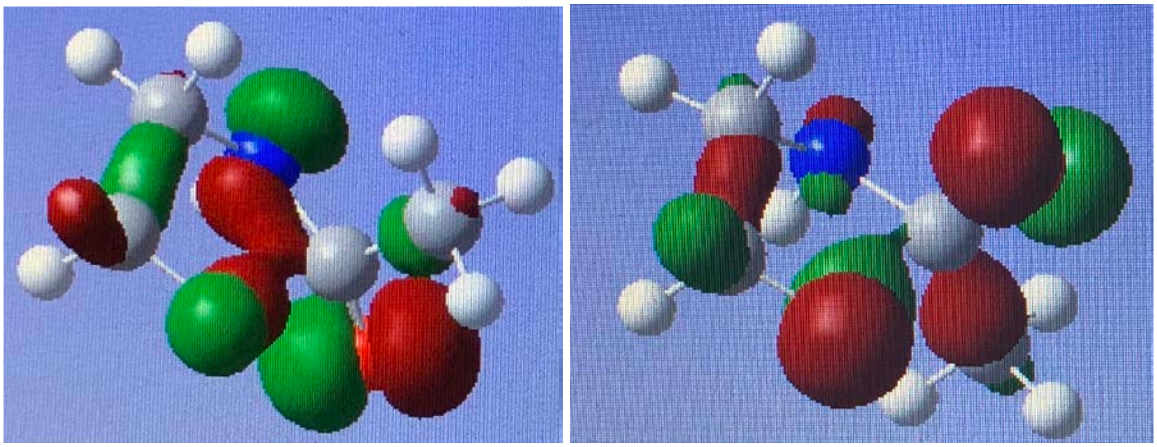

Figure S2. Calculated HOMO-1 contour surfaces for **2cis‡** (X=O) and **2trans‡** (X=O), showing better O···C overlap for cis (red) than for trans (green).
